# Wake Up, Chancellor! Business First

**Published:** 1913-01-25

**Authors:** 


### The Hospital

The Workers' Newspaper of Administrative Medicine and Institutional Life, Administration, National Insurance and Health.

No. 1386, Vol. LIII. SATURDAY, JANUARY 25, 1913. PRICE ONE PENNY.

### WAKE UP, CHANCELLOR! BUSINESS FIRST.

It becomes increasingly plain that but for the voluntary hospitals it would be impossible for the National Insurance Act, as it stands, to be worked successfully or at all as matters are at present. It will take, no doubt, much forbearance on the part of all the hospitals, and the exhibition of much greater readiness and generous consideration to meet the views, wishes, and necessities of insured persons on the part of those immediately responsible for the working of the National Insurance Act, to enable this great change in our national system of medical relief to be brought into being and made acceptable to the people. At present everybody is overburdened, and the majority of insured persons needing medical benefit are subjected to many regrettable risks, with the loss of much valuable time. These evils could have been avoided had more wisdom and knowledge been displayed by the Government in regard to the construction, machinery, and working of this measure in its initial stages. No blame can properly attach to the National Health Insurance Commissioners and their staff, who had imposed upon them a well-nigh impossible task, which the exhibition of a little statesmanship resting on experience should have readily prevented. We recognise that Mr. Lloyd George possessed a genuine desire to help the least fortunate and humbler folk to the ready attainment of adequate medical treatment when ill. Yet any amount of sympathy, apart from foresight and statesmanship, could not have prevented all the present failures and difficulties which have arisen. The cause lies in the absence of administrative ability, for neither knowledge nor experience were exhibited on the introduction of this measure of National Insurance.

We are wishful that these mistakes may be frankly recognised by Mr. Lloyd George, and that he will now drop politics and stick closely to business. The circumstances and associations of our daily life and work make us acutely familiar with the really appalling weight of care, and the infinite risks which at present oppress hundreds of the most deserving and thrifty of insured persons in every great centre of population throughout the United Kingdom. The majority of these poor but honest people have been ruthlessly deprived by the regulations of the comfort and strength which they possessed previous to the 15th instant by commanding the services of the practitioner whom they knew and trusted, and who had an intimate knowledge of their constitution and family circumstances. They have been compulsorily insured; they have had to, and still, pay willy-nilly a weekly sum for medical benefit, and now when they are ill they find themselves deprived of their medical attendant without rhyme or reason that they can understand. Further, they are made to face the loss of time and the worry of crushing their way through a crowd of people in order to try and obtain some doctor with a name familiar to them residing within reach whom they may consult when they find themselves ill or in need of medical treatment at home. Would not Mr. Lloyd George himself and Mrs. Lloyd George refuse to be deprived in the day of illness of the strength and consolation which the attendance of their own doctor secures to them? If Mr. George is ever to be heard of as a statesman it is essential that, either by his own administrative gifts, or by supplementing them by the best administrative gifts he can secure, the present cruelties of National Insurance shall be lifted from the shoulders of the insured without a moment's avoidable delay. We have only taken one of many equally grave hardships caused by needlessly exasperating regulations and difficulties which confront insured persons at the present moment. Could the malignest imp of mischief, being in fact the bitterest foe of Mr. Lloyd George, have conceived a plan better calculated to make the Chancellor appear to the classes who represent the very backbone of the English people as a man without feeling who is more wantonly cruel than words can describe?

Last week we published a verbatim report of Lord Crewe's interesting speech upon National Insurance and the voluntary hospitals. We welcome that speech because it indicates that at the bottom, strange as it may appear to the supporters and managers of voluntary hospitals throughout the country, the Government, or the majority of them, do desire to perpetuate the voluntary system of hospitals. The economist, no doubt, will say: "Granted that they do, there is nothing to thank them for, seeing that no Government could exist which attempted to add twenty millions to our present national expenditure by destroying the voluntary hospitals." No doubt the economist would be right in the economical sense, but our point is that the awful administrative muddles, and the dangers which threaten insured persons through them at the moment, render it of the first importance that the Government should be awake to the absolute necessity of preventing by foresight and immediate action any possible risk of a breakdown in our present hospital system. Were such a breakdown to follow as a result of National Insurance the calamity to the working classes and to hundreds of thousands of worthy but humble people up and down the country might prove as great as that resulting from a raging pestilence passing over these islands from end to end. This is another reason why in all sympathy with the difficulties in which he finds himself we most earnestly urge Mr. Lloyd George to face the hospital position quietly and at once prepare to meet any contingency which may arise as and when occasion may offer. We have urged this point upon the National Health Insurance Commissioners, upon the Chancellor of the Exchequer, and upon the Government. Lord Crewe stated he was certain that the plan we have propounded in these columns to obviate the risk of any hospital breakdown, whilst safeguarding the voluntary system, is one which will receive the very closest and most careful consideration from the department especially concerned, and ultimately the close attention and examination of the Government, should necessity occur. Lord Crewe admits that, if we hospital people generally had regarded the Insurance Act in its operation as hostile to the hospitals, it would have been a severe blow to the Government. Seeing that the Government have cast upon the voluntary hospitals the whole cost and burden of treating all serious cases of illness and accident and all cases of special disease which may occur amongst insured persons, free of all charge to the Government, it is the Government's plain duty to set up the simple machinery requisite under expert guidance to keep the National Health Insurance Commissioners in touch with the hospital position and to enable the Treasury to prepare for and be ready to meet any emergency which may arise. Self-preservation is the first law of Nature, and to be the protector and friend of the humblest, especially when ill, is the declared aim of the present Chancellor of the Exchequer. Wake up, Mr. Lloyd George!

